# Immunology of pregnancy and sepsis: shared and specific pathways guiding future precision care

**DOI:** 10.1016/j.ebiom.2026.106364

**Published:** 2026-07-11

**Authors:** Harindra E. Amarasinghe, Julian C. Knight, Marian Knight

**Affiliations:** a Chinese Academy of Medical Sciences Oxford Institute, University of Oxford, Oxford, UK; b National Perinatal Epidemiology Unit, Nuffield Department of Women's and Reproductive Health, University of Oxford, Oxford, UK; c Centre for Human Genetics, Nuffield Department of Medicine, University of Oxford, Oxford, UK; d John Radcliffe Hospital, Oxford University Hospitals NHS Foundation Trust, Oxford, UK

**Keywords:** Pregnancy, Sepsis, Shared, Maternal sepsis, Precision medicine, Future directions

## Abstract

Maternal sepsis remains a leading cause of preventable death globally, yet its underlying biology within the context of pregnancy is poorly understood. This review examines the extent to which pregnancy and sepsis share physiological, immunological, and regulatory pathways. We propose a unifying model for maternal sepsis that integrates immune, transcriptomic, epigenetic, and metabolic frameworks to advance a precision medicine approach, restoring immune equilibrium, controlling pathological inflammation while preserving pregnancy-specific adaptations. We address key knowledge gaps in current precision maternal care in sepsis and discuss the potential and limitations of emerging approaches supporting individualised risk assessment, trimester-specific immune monitoring, and targeted recovery interventions. Pregnancy itself serves as a model for natural immunological equilibrium, where tolerance and defence are finely balanced, offering broader insights for sepsis management beyond obstetrics. Translating these insights into equitable, pregnancy-compatible therapeutic strategies is an exciting opportunity to reduce the global burden of maternal sepsis and improve outcome.

## Introduction

Maternal sepsis, a life-threatening organ dysfunction condition resulting from infection during pregnancy, childbirth, post-abortion, or the postpartum period,^1^ remains a leading cause of preventable morbidity and mortality globally, accounting for 11% of direct maternal deaths^2^ and an estimated 19 million cases in 2021.^3^ This figure however, likely underrepresents the true burden due to inconsistent case definitions, limited surveillance and underreporting, particularly in low- and middle-income countries (LMICs), where data remain sparse. Despite variations in global data, it is estimated about 70 women per 1000 live births require hospital care for infection, and 11 experience severe infection-related outcomes.^2^ The World Health Organisation Global Maternal Sepsis Study reported that over half of intrahospital maternal deaths were infection-related, with maternal infection or sepsis contributing to 8–12% of obstetric intensive care admissions globally.^2^ While LMICs bear the greatest burden, high-income countries are not exempt: infection remains the second leading cause of maternal death in both the United Kingdom and the United States.^2^^,^^4^, ^5^, ^6^, ^7^

Beyond its contribution to maternal mortality, sepsis during pregnancy or postpartum is associated with substantial long-term morbidity.^7^ In the UK, for every maternal death from sepsis, about 50 women experience life-threatening severe sepsis.^8^ Sepsis survivors, regardless of pregnancy status, face poor long-term outcomes, including recurrent sepsis and chronic inflammatory sequelae.^9^ Following sepsis during pregnancy, these consequences extend to neonatal and child health, with increased risks of stillbirth, preterm birth, foetal growth restriction, and adverse early childhood development.^10^ Moreover, socioeconomic deprivation, structural inequities, and ethnic minority status are consistently linked to higher maternal sepsis risk across diverse health systems, reflecting both biological and systemic vulnerabilities.^11^

A critical barrier to addressing these challenges is the limited understanding of sepsis pathophysiology and the tendency for its manifestations to be misinterpreted in pregnancy.^12^ Highly dynamic trimester-specific physiological and immune adaptations complicate timely recognition of sepsis, reduce the sensitivity to conventional sepsis diagnostic tools, and hinder effective treatment.

This review explores sepsis pathophysiology and pregnancy biology as distinct yet interrelated processes, analysing overlapping physiological, immunological, and regulatory landscapes, and the divergent outcomes that emerge at their intersection. We dissect the dynamic interplay between immune cell modulations and key regulatory pathways (including transcriptional, epigenetic, metabolic, and proteomic), emphasising their dual roles across gestation and in sepsis. We propose that identifying the points of intersection between these contexts can inform valuable insights to achieve immunological equilibrium, limiting pathological inflammation while preserving pregnancy-specific adaptations, with direct implications for advancing maternal sepsis care. Given the limitations of universal approaches, we highlight the therapeutic importance of accounting for inter-individual heterogeneity in maternal immune states, and outline emerging precision medicine strategies, including personalised cytokine and transcriptomic profiling, targeted immunomodulation, epigenetic and metabolic interventions, and artificial intelligence-driven decision support that can be tailored to this shared regulatory landscape. Together these approaches lay the groundwork for developing future tools, with the potential to enable timely, safe, and pregnancy-compatible care. Finally, we suggest that the pregnancy-associated immune adaptations, marked by temporal immune shifts, transcriptional balance, and controlled inflammation, may provide a model of natural immune recalibration. A deeper understanding of this model may offer key insights into how the immune system navigates inflammatory extremes without collapsing into dysfunction, guiding the development of phase-specific, personalised therapeutic strategies for sepsis beyond pregnancy.

## Intersection of physiological shifts in pregnancy that mask, mimic, and modify sepsis presentation

**Fig. 1:**
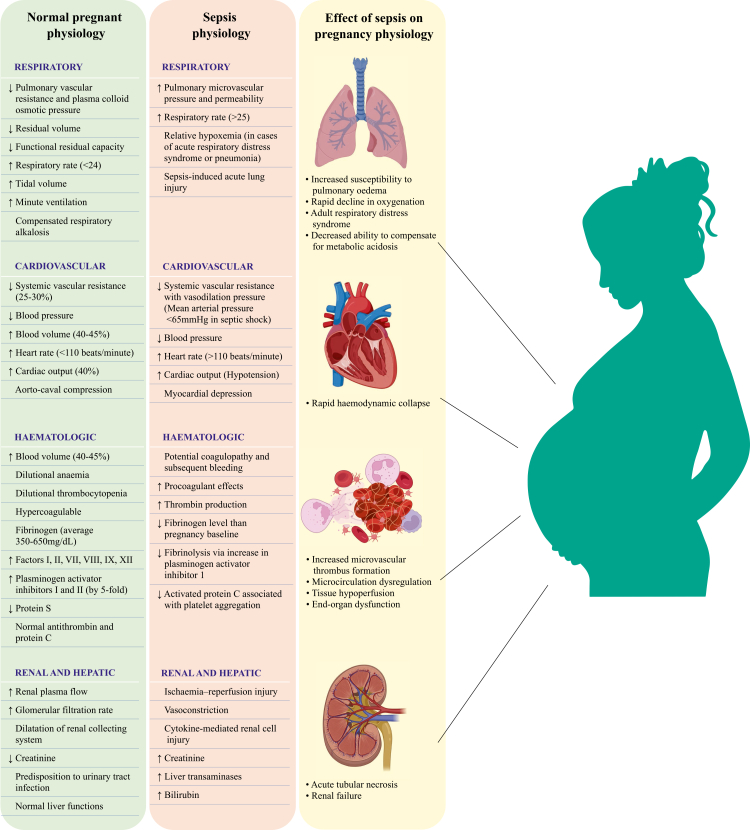
**Comparative overview of physiological changes during normal pregnancy, sepsis, and the impact of sepsis on maternal physiology**.^13^, ^14^, ^15^

Normal pregnancy leads to physiological adaptations across the cardiovascular, respiratory, renal, and haematologic systems, changes that overlap strikingly with those seen in sepsis (Fig. 1).^13^, ^14^, ^15^, ^16^ In pregnancy, they serve adaptive functions, supporting foetal growth and maintaining maternal homoeostasis. In sepsis however, similar responses become pathological, driving tissue hypoperfusion and multi-organ dysfunction. During pregnancy, systemic vascular resistance and blood pressure fall with compensatory increases in blood volume, heart rate, and cardiac output whereas in sepsis, similar vasodilation occurs pathologically, accompanied by myocardial depression and reduced perfusion. Haematologic changes in pregnancy promote a hypercoagulable state, while sepsis disrupts coagulation control, predisposing to coagulopathy and bleeding. Gestational adaptations may therefore, either mimic or obscure early manifestations of sepsis, delaying diagnosis.^12^

The immediate postpartum period further complicates diagnosis, as physiological inflammatory responses to labour and tissue injury mimic sepsis.^17^ Even without infection, during labour and early postnatally, C-reactive protein rises markedly, while procalcitonin, usually low in pregnancy, may also rise transiently, reducing the specificity of conventional sepsis biomarkers.^15^^,^^18^ Moreover, the adaptations that sustain pregnancy may reduce maternal physiological reserve, limiting the capacity to compensate for infection-induced cardiovascular, respiratory, and renal stress, and thereby intensify severity in certain infections.^15^ For example, pregnant women have a 2·4-fold higher risk of hospitalisation from influenza compared to non-pregnant women of reproductive age with influenza,^19^ and experienced worse outcomes during the 2009 H1N1^20^ and COVID-19 pandemics than non-pregnant women.^21^ Pregnancy also raises susceptibility to specific infections such as malaria, listeriosis, and urinary tract infections, with a three-fold higher risk of severe malaria^22^^,^^23^ and 20-fold higher risk of invasive group A streptococcal infection postpartum, compared with non-pregnant women.^8^

## Intersection of immunological landscapes in pregnancy and sepsis

**Fig. 2:**
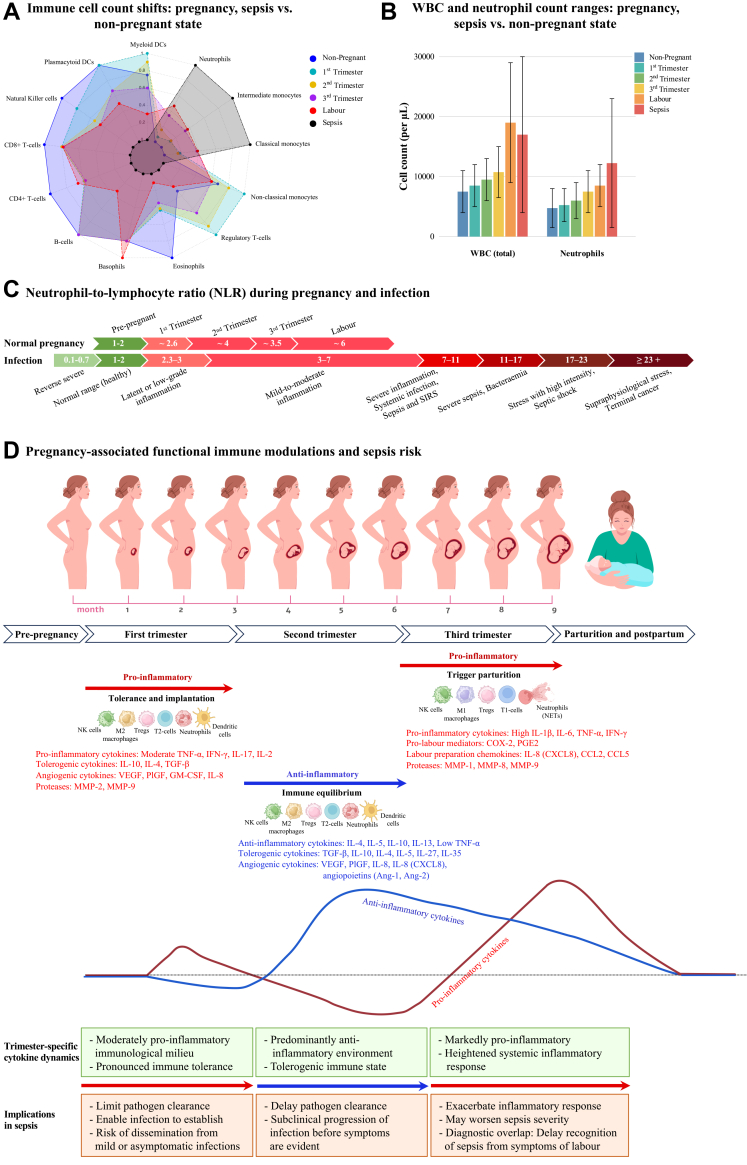
**Immunological landscape of a normal pregnancy and sepsis pathology, in comparison to non-pregnant status**. Modulations in immune cell counts: (A) A radar plot illustrating the shifts in immune cell counts across pregnancy trimesters, at parturition, and in sepsis, relative to pre-pregnant levels. Values are normalised to a 0–1 scale representing relative immune cell abundance in each state. The plot is derived from reported mean immune cell counts in healthy pregnancy and sepsis from published literature. (B) Range of variation in white blood cell (WBC) and neutrophil counts across pregnancy, labour, and sepsis compared with the non-pregnant state. (C) Changes in innate and adaptive inflammatory balance as reflected by the Neutrophil-to-Lymphocyte Ratio (NLR), during a normal pregnancy compared to sepsis in the non-pregnant state.^24^, ^25^, ^26^ Modulations in immune cell functions: (D) Functional adaptations of the immune system during pregnancy, representing trimester-specific cytokine and inflammatory dynamics, pertinent immune cell types involved, and their implications in sepsis. Data (normal pregnancy^14^^,^^24^, ^25^, ^26^, ^27^, ^28^, ^29^, ^30^, ^31^, ^32^, ^33^, ^34^, ^35^, ^36^, ^37^, ^38^, ^39^, ^40^, ^41^ and non-pregnant sepsis^9^^,^^21^^,^^23^^,^^24^^,^^28^^,^^30^^,^^33^, ^34^, ^35^^,^^38^^,^^42^, ^43^, ^44^, ^45^, ^46^, ^47^, ^48^, ^49^, ^50^, ^51^) underpinning the figures are available in Supplementary Table S1.

During pregnancy, the immune system dynamically adapts to maintain equilibrium. Sepsis represents a maladaptive host response to infection, where ordinarily protective immune mechanisms become dysregulated, causing systemic inflammation, immune suppression, and organ dysfunction. The immune shifts shared between the two states are reflected in immune cell counts (Fig. 2A–C, Supplementary Table S1) and their functional profiles (Fig. 2D), and may provide key mechanisms underlying the heightened risk of sepsis during pregnancy.

## Dynamics of immune cell counts during pregnancy

Although immune cell counts change across pregnancy, these shifts are typically modest, remain near healthy non-pregnant reference ranges (Fig. 2A), and largely reflect peripheral blood (systemic) changes. Physiological leucocytosis develops with advancing gestation, rising further during labour and early postpartum,^27^^,^^28^^,^^52^ driven predominantly by a progressive increase in neutrophil count, reflecting physiologically balanced, non-toxic neutrophilia (Fig. 2B).^28^ Monocyte subsets shift, with modest increases in classical monocytes, expansion of intermediate monocytes in mid-to-late gestation, and slightly reduced non-classical monocytes.^29^^,^^30^ A mild, regulated lymphopenia supports foetal tolerance; lymphocyte counts decline early but remain within physiological limits and recover in the third trimester.^28^^,^^31^ Eosinophils fall transiently around labour and rebound postpartum (days 3–5).^32^ Basophils show a mild second-trimester decline with recovery to non-pregnant levels by early puerperium. Mild gestational thrombocytopaenia is common, typically remaining within or near the lower limit of normal.^33^

The neutrophil-to-lymphocyte ratio (NLR) reflects the systemic balance between innate and adaptive immune responses, providing a real-time index of immune homoeostasis across physiological and pathological states. In healthy non-pregnant adults, the NLR typically ranges from 1–2.^24^ During normal pregnancy, the NLR rises modestly in the absence of infection and increases further during labour (range 3–6, Fig. 2C).^25^ These changes reflect mild, physiological inflammation. Maternal complications and infection such as preeclampsia and chorioamnionitis are associated with higher values (around 10).^26^

## Trimester-specific functional immune adaptations in pregnancy

Pregnancy is characterised by a finely regulated, dynamic cytokine environment that follows a trimester-specific pattern, alternating between pro-inflammatory and anti-inflammatory states (Fig. 2D).

### First trimester

Early pregnancy is characterised by a tightly regulated, predominantly localised pro-inflammatory milieu at the uterine–placental interface (Fig. 2D). Decidual natural killer (dNK) cells (∼70% of decidual leukocytes) are key regulators of early placentation. Unlike peripheral NK cells which mediate cytotoxic defence against infected cells, dNK cells are non-cytotoxic, secreting angiogenic and immunomodulatory factors such as Vascular Endothelial Growth Factor (VEGF), Placental Growth Factor (PlGF), Interleukin (IL)-8 and tightly regulated IFN-γ, to facilitate trophoblast invasion, decidualisation, spiral artery remodelling and the establishment of maternal–foetal tolerance.^34^ Neutrophils contribute to implantation via matrix remodelling, secreting matrix metalloproteinases (MMP-9).^35^ Classical monocytes expand but remain functionally restrained, producing moderate Tumour Necrosis Factor alpha (TNF-α) and IL-6 to sustain the pro-inflammatory milieu without excessive inflammation.^29^ Decidual macrophages acquire an M2-like, tolerogenic profile. Adaptive immunity shifts toward tolerance, with reduced Th1/Th17 responses, increased Th2 cytokines (IL-4, IL-5, IL-10, IL-13), expansion of regulatory T-cells (Treg; from 4·4% pre-gestational level to 6·7% of the total peripheral lymphocyte population), tolerogenic dendritic cells, and IL-10–producing regulatory B-cells.^36^^,^^37^ Together, these coordinated adaptations maintain the balance between controlled local inflammation, and immune tolerance to sustain early pregnancy while systemic immune modulations remain comparatively subtle.

### Second trimester

The immune system shifts toward an anti-inflammatory state that sustains maternal–foetal tolerance and supports placental and foetal growth.^38^ As placental development stabilises, dNK cells begin to decline.^34^ Neutrophils gain regulatory functions, secreting IL-10 and cooperating with Tregs to maintain immunosuppression.^35^ Intermediate monocytes expand and contribute to placental maturation.^39^ Macrophages adopt M2-like anti-inflammatory phenotypes but retain plasticity to switch to a pro-inflammatory M1-phenotype in response to pathogens.^39^ Dendritic cells promote Treg differentiation. Adaptive immunity remains skewed toward a Th2 dominant tolerant phenotype, suppressed Th1/Th17 responses, and continued Treg expansion occurs.^36^^,^^37^ Low levels of pro-inflammatory cytokines help in preserving immune surveillance, but their excess is linked to complications such as preeclampsia, preterm birth and intrauterine growth restriction.^53^ Overall, the second trimester is defined by immune equilibrium, balancing defence with tolerance to enable sustained foetal development (Fig. 2D).

### Third trimester

The maternal immune system shifts back to a pro-inflammatory state, to prepare for labour while retaining residual tolerance to the foetus.^38^ dNK cells decline further but secrete IFN-γ and TNF-α to promote uterine contractility and tissue remodelling.^34^ Neutrophils increase in number (Fig. 2B), and activation occurs both locally and systemically. Recruited by IL-8 to gestational tissues, they release pro-inflammatory cytokines, prostaglandins, and MMPs, driving cervical ripening, membrane rupture, and myometrial activation.^35^ Moderate neutrophil extracellular traps (NETs) formation enhances antimicrobial defence, though excess is linked to complications such as preeclampsia which highlights the importance of immune balance for a successful pregnancy.^35^ Macrophages polarise towards a pro-inflammatory M1 phenotype,^39^ amplifying inflammatory cascades. Treg frequency and their suppressive functions decline.^37^ Th1 and Th17 responses necessary for labour, re-emerge while systemic lymphocyte counts begin to normalise.^36^ CD8^+^ T-cells regain cytotoxicity, contributing to pro-inflammatory signalling. Activated dendritic cells further promote pro-inflammatory T-cell responses. B-cells remain rare in the decidua but mediate transplacental maternal–foetal antibody transfer.^36^ Collectively, the third trimester is marked by immune reactivation that orchestrates parturition while balancing host defence and foetal protection until delivery (Fig. 2D).

Maintenance of a healthy pregnancy thus relies on finely tuned immunological adaptations that follow a precise and predictable sequence, often referred to as the “immune clock of pregnancy”.^54^

## Immune cell count variations in sepsis

Relative to physiological pregnancy, sepsis is characterised by more pronounced alterations in systemic immune cell counts, often exceeding established healthy reference ranges (Fig. 2A). Early sepsis may present with leucocytosis or leukopenia, though values vary with age, pathogen, and disease severity, and counts may reach as high as 30 × 10^3^/μL in infections such as *Streptococcus pneumoniae*.^42^ In maternal sepsis, white blood cell (WBC) counts <4 × 10^3^/μL or 15–30 × 10^3^/μL may indicate pathology; however, these ranges overlap substantially with physiological postpartum leucocytosis and labour-related stress responses.^43^ Neutrophil counts similarly show high variability (Fig. 2B), with early neutrophilia beyond physiological limits followed by neutropenia (<1500/μL), or profound neutropenia (<500–1000/μL), indicating severe haematopoietic dysfunction.^55^ Profound lymphopenia involving CD4^+^, CD8^+^ T-cells, and B-cells (Fig. 2A), driven by apoptosis and impaired lymphopoiesis is a hallmark of sepsis.^44^ In non-pregnant populations, NLR values > 3·0 or <0·7 suggest moderate inflammation (Fig. 2C) while in severe pathology including sepsis, values exceed 11–17 and may rise above >23 in septic shock or polytrauma (Fig. 2C)^24^ although thresholds can vary with demographic and lifestyle factors.^25^

Classical monocytes initially expand to support phagocytosis but later become functionally exhausted. Intermediate monocytes, key mediators of antigen presentation and cytokine production, increase markedly (Fig. 2A) and correlate with disease severity, while non-classical monocytes specialised for vascular surveillance, decline.^45^ Emergency myelopoiesis drives rapid expansion and premature release of neutrophils and monocytes, producing a dysregulated “left shift” compared to the controlled myeloid adaptation seen in pregnancy.^46^ This may temporarily enhance pathogen clearance but risks immune dysregulation and bone marrow exhaustion if sustained. Monocyte distribution width increases with sepsis severity and pregnancy complications,^47^ may reflect immune-metabolic programming involving glycolysis, lactate signalling, and fatty acid oxidation.^56^ Dendritic subsets decline modestly during pregnancy but profoundly depleted in sepsis (Supplementary Table S1).^40^ Sepsis also causes marked thrombocytopaenia, which correlates with shock, multi-organ dysfunction, and mortality.^33^

## Functional immune shifts in sepsis pathophysiology

Immune dysregulation in sepsis is complex and dynamic, with pro-inflammatory and anti-inflammatory responses occurring concurrently rather than in distinct sequential phases.^57^^,^^58^ Following pathogen recognition, a rapid inflammatory cascade is initiated, characterised by a surge in pro-inflammatory cytokines (TNF-α, IL-6, and IL-1β), leading to microvascular thrombosis, increased vascular permeability, capillary leak, shock, and subsequent organ dysfunction.^48^ NETs further amplify inflammation through sustained cytokine release.^49^ Simultaneously, compensatory anti-inflammatory pathways are activated in an attempt to restore immune homoeostasis. However, an excessive or sustained anti-inflammatory response may result in immunoparalysis, increasing the susceptibility to secondary infections and adverse clinical outcomes. In this context, monocytes undergo functional reprogramming toward an endotoxin-tolerant phenotype. Elevated IL-10 and TGF-β levels, lymphocyte apoptosis, T-cell exhaustion (mediated by *PD-1*), reduced T-cell receptor diversity, diminished cytotoxic function, and impaired antibody responses collectively weaken adaptive immunity, leading to profound immune dysfunction.^57^^,^^58^

### Postnatal and post-sepsis immune cell recovery

Following both pregnancy and sepsis, immune cell counts gradually return toward baseline, reflecting resolution of acute physiological stress and restoration of homoeostasis. Pregnancy-related leucocytosis may persist for 6–8 weeks postpartum,^28^ with WBC counts returning to antenatal levels by day 7 and declining to non-pregnant ranges by ∼3 weeks.^52^ Neutrophil and monocyte counts typically normalise within 4 weeks,^41^ and platelet counts within 12 weeks.^28^ In contrast, recovery after sepsis is often prolonged and numerical normalisation does not necessarily reflect restored immune function.^9^ Lymphopenia usually resolves within one month, but functional T-cell recovery may take several months,^41^ Persistent day 4 lymphopenia after sepsis is associated with increased short- and long-term mortality, reflecting impaired host defence,^44^ and many survivors develop persistent immunoparalysis with increased susceptibility to secondary infections and viral reactivation.^9^

## Intersection of pregnancy-associated and sepsis-induced functional immune modifications

**Fig. 3:**
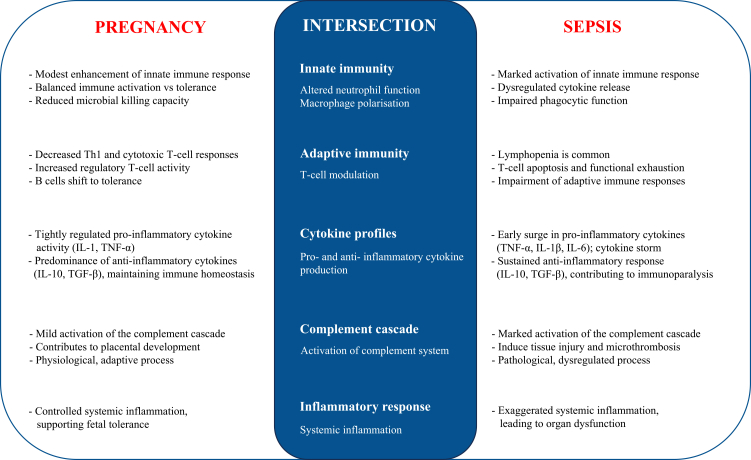
**Shared functional immunological landscape of pregnancy and sepsis**.

Underlying the two distinct entities of pregnancy and sepsis, lie overlapping functional immune adaptations (Fig. 3). Both involve innate immune alterations, including neutrophil activity and macrophage polarisation, as well as modulation of adaptive immunity such as T-cell responses. These cellular changes are accompanied by shifts in cytokine profiles, systemic inflammatory responses, and complement system activity, which are physiologically regulated in pregnancy but pathologically activated in sepsis.

In maternal sepsis, such overlapping immune modifications may contribute to adverse pregnancy outcomes. In early pregnancy, localised pro-inflammatory activity at the maternal–foetal interface coexists with relatively mild systemic immune modulation, and may limit pathogen clearance allowing infections to establish before clinical symptoms are evident. During mid gestation, the predominantly anti-inflammatory, tolerogenic systemic environment may further dampen immune response, enabling subclinical progression of infection. Maladaptive placental response, shifting from a tolerogenic barrier to a pro-inflammatory organ, and imbalance in angiogenic mediators (e.g., VEGF and PlGF) promote maternal vascular malperfusion and placental hypoxia.^34^^,^^38^ At term, physiological inflammation during labour can mask or mimic sepsis, complicating diagnosis; when compounded by sepsis-induced hyperinflammation, disease severity may be amplified, exceeding levels observed in non-pregnant individuals (Fig. 2D).^38^ For instance, reduced peripheral NK cell numbers in the third trimester associated with compensatory increase of cytokine production in response to influenza A contributes to more severe disease.^50^ Additionally, maternal infection can trigger a foetal inflammatory response syndrome via transplacental inflammatory signalling, raising risks of neurodevelopmental injury and neonatal death.^34^

## Regulatory convergence in pregnancy and sepsis: shared regulatory pathways

**Fig. 4:**
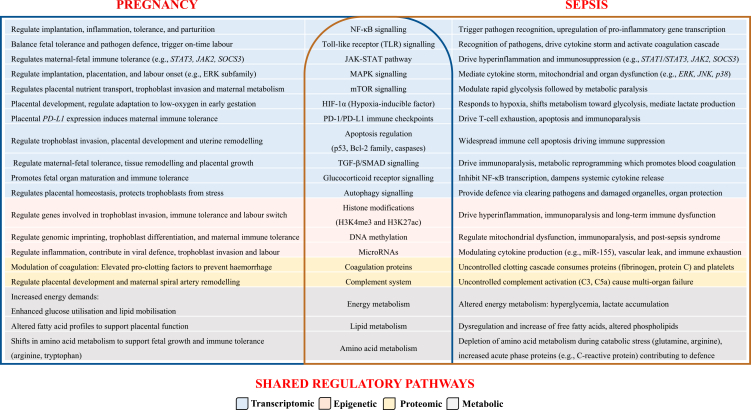
**Shared regulatory pathways and their dual roles played in pregnancy and sepsis**.^9^^,^^21^^,^^23^^,^^30^^,^^34^^,^^38^^,^^48^, ^49^, ^50^, ^51^^,^^56^^,^^58^, ^59^, ^60^, ^61^, ^62^, ^63^, ^64^, ^65^, ^66^, ^67^, ^68^, ^69^, ^70^, ^71^, ^72^, ^73^, ^74^, ^75^, ^76^

The functional immunological convergence of pregnancy and sepsis is underpinned by overlapping transcriptional, epigenetic, proteomic, and metabolomic regulatory networks (Fig. 4). Yet the context, timing, and magnitude of pathway activation differ profoundly.

### Transcriptional regulation

Key transcriptional pathways such as Nuclear Factor kappa-light-chain-enhancer of activated B-cells (NF-κB), Janus Kinases and Signal Transducers and Activators of Transcription (JAK–STAT), Mitogen-Activated Protein Kinase (MAPK), NOD-like receptor protein 3 (NLRP3), and type I interferon (IFN) signalling are active in both pregnancy and sepsis. JAK–STAT, MAPK and NLRP3 govern cytokine-mediated immune responses, while IFN signalling balances immune activation and tolerance.^38^^,^^51^

NF-κB is a central regulatory node in both pregnancy and sepsis, but its role diverges across the two states. In pregnancy, its activity is tightly and temporally regulated to support gestation: early gestation features controlled NF-κB–driven pro-inflammatory cytokines (IL-1β, IL-6, IL-8, TNF-α, IFN-γ, IL-12, IL-23) and chemokines (CCL2, CCL5) to support implantation and decidualisation; mid-gestation suppresses NF-κB activity promoting tolerance via anti-inflammatory mediators (progesterone, IL-10, TGF-β); near term, NF-κB reactivation triggers inflammation (IL-6, IL-8, TNF-α, MMPs) that promotes uterine contractions, membrane rupture, and labour.^59^ In sepsis, NF-κB activation is dysregulated: following Toll-like receptor (TLR) 4-mediated pathogen recognition, NF-κB drives sustained, excessive production of pro-inflammatory mediators, leading to cytokine storm, immune exhaustion, vascular injury, and multi-organ dysfunction.^48^

As with NF-κB, TLR signalling and NLRP3 inflammasomes are tightly regulated in pregnancy to balance foetal tolerance and pathogen defence, avoiding excessive inflammation.^38^ In sepsis, their dysregulation leads to pro-inflammatory cytokine production, and widespread systemic inflammation. Aberrant TLR activation has been implicated in obstetric complications such as preterm birth and pre-eclampsia.^38^^,^^60^

### Epigenetic regulation

Similarly, pregnancy and sepsis share epigenetic mechanisms (histone modifications and DNA methylation) that modulate inflammatory gene expression critical for immune activation.^23^ In both conditions, microRNAs such as miR-146a and miR-155 regulate TLR signalling and NF-κB activation, shaping the magnitude and duration of immune responses.^61^

### Proteomic overlap

Proteomic analyses in both pregnancy and sepsis reveal activation of complement and coagulation cascades, upregulation of acute-phase proteins, induction of stress-response, and heat shock proteins, reflecting systemic inflammation and immune stress. In normal pregnancy, these responses are tightly regulated, whereas in complications such as preeclampsia and sepsis, they are dysregulated.^53^

### Metabolic adaptations

Both states involve cellular metabolic reprogramming, including enhanced glycolysis, modulation of the tricarboxylic acid cycle, and altered lipid and amino acid metabolism, which supports immune signalling and energy balance.^62^ However, pregnancy represents a physiological metabolic adaptation with programmed insulin resistance to meet maternal–foetal demands, whereas sepsis induces a pathological hypermetabolic, catabolic state marked by mitochondrial dysfunction, energy failure, and tissue breakdown.^56^^,^^63^

In maternal sepsis, multi-omics studies show that although pregnancy is tightly regulated, it remains an immunologically plastic state that can be reshaped by inflammatory stress. Infection with SARS-CoV-2 provides a clear example of this reprogramming: placental trophoblasts and decidual immune cells show upregulated interferon-stimulated genes (e.g., *IFIT1, ISG15*) and NF-κB–regulated cytokines (e.g., IL6, TNF, CXCL10), alongside dysregulated vascular remodelling (e.g., *VEGFA, MMP9*), reflect the transcriptional activation of antiviral and pro-inflammatory pathways. Elevated proteomic evidence further demonstrates that immune activation extends beyond inflammation, disrupting endothelial and structural homoeostasis, with altered foetal immune signatures detectable in cord blood even without vertical transmission.^21^ Infection also induces substantial maternal epigenomic changes via retrotransposon dysregulation,^64^ and enhanced foetal stress haematopoiesis.^65^

A deeper understanding of the shared regulatory networks of pregnancy and sepsis, and the roles they play in each context offers valuable insight for future therapeutic directions in maternal sepsis. Defining an immunological equilibrium within this shared space, where sepsis-associated pathological inflammation is restrained while physiological pregnancy adaptations are preserved, represents a critical goal for advancing future maternal sepsis research.

However, this immune equilibrium is highly individualised, and shaped by a complex interplay among the host (genetic background, age, ethnicity, metabolic status), foetus (paternal antigens), pathogen (virulence, load, resistance), environment (microbiome, delivery setting, and community context) and pregnancy-specific (gestational age, maternal hormones) factors, and also shifts dynamically across the sepsis continuum. In this context, precision medicine provides a framework to unravel these complex immune trajectories in a personalised and temporally informed manner.

## Emerging directions and precision medicine therapeutic targets for maternal sepsis

Maternal sepsis poses unique challenges compared with sepsis in non-pregnant populations. The maternal immune baseline is dynamic across trimesters, and any intervention must consider both maternal and foetal outcomes. In severe, refractory cases where standard therapy fails, balancing benefit and risk is critical, demanding the need for deeper insights to guide treatment decisions. Yet evidence for targeted therapies in pregnancy remains limited, with strategies often extrapolated from non-pregnant populations. Against these challenges, emerging precision medicine approaches offer the potential to better characterise sepsis in pregnancy, and guide tailored, phase-specific therapies. It is important to recognise that in the context of the critical illness of maternal sepsis, potential benefits will outweigh theoretical harms of many therapies, as recognised in the recently updated WHO guidance for best practices for clinical trials.^77^

### Immune profiling and personalised cytokine signature

Pro-inflammatory cytokines such as IL-1β, CCL4, CCL5, and CXCL10 are emerging as potential biomarkers of sepsis severity and cytokine storm.^48^^,^^66^^,^^67^ Personalised cytokine signatures and immune profiling accounting for pregnancy-specific immune shifts, may accurately predict disease trajectory and guide real-time, targeted interventions. Multiplexed immune profiling platforms for circulating and intracellular cytokines such as Luminex and flow cytometry support such patient stratification, enabling delivery of therapies to those most likely to benefit. Comparing cytokine patterns with trimester-matched controls^68^ may provide baseline information to tailor the pathways requiring modulation: patients with hyperinflammatory profiles (elevated TNF-α, IL-1β, IL-6) may benefit from cytokine suppression, whereas those with immune-exhaustion (low HLA-DR, high IL-10) may require stimulatory therapies such as GM-CSF.^48^ However, evidence supporting personalised immune profiling in pregnancy remain limited, highlighting a critical gap in current research.

### Transcriptomic profiling, genetic screening and targeting dysregulated immune checkpoints

Transcriptomic profiling provides dynamic, high-resolution snapshots of maternal immune states, enabling patient-specific characterisation of sepsis risk, disease trajectory, and therapeutic responsiveness.^66^ While transcriptomic endotypes such as Sepsis Response Signatures predict outcomes in non-pregnant sepsis,^69^ their translation to maternal sepsis requires prospective validation, accounting for trimester-specific adaptations, placental signalling, and the unique immunological and hormonal milieu of pregnancy.

Key regulatory pathways such as NF-κB, JAK–STAT, and IFN signalling offer promising precision medicine targets for selective inhibition or temporal modulation.^60^ Early pregnancy interventions might focus on transient dampening of hyperinflammation (for example NF-κB modulation, or low-dose IL-2-mediated Treg enhancement), while mid-pregnancy approaches may aim to restore immune competence and vascular integrity such as IFNγ and mitochondrial support. Late-gestation, when immune activation primes labour, therapies may balance infection control with tissue protection through strategies targeted on NETs (such as DNase, *PAD4* inhibitors),^49^ inflammasome-targeted therapy^70^ or maternal gene delivery,^71^ though the evidence of benefit versus risk in pregnancy remains limited.

Genetic variants, such as TLR4 polymorphisms, human leucocyte antigen (HLA), proteasome subunit alpha type-4 expression level^72^ may aid risk stratification, though distinguishing adaptive maternal–foetal tolerance from pathological immunosuppression is essential. For instance, reduced monocyte HLA-DR expression indicates immunosuppression in non-pregnant sepsis whereas physiological downregulation of specific HLAs is critical in maternal–foetal tolerance.^73^ Emerging single-cell, spatial, and long-read sequencing, now allow detailed, cell-, tissue-, isoform-, and allele-specific transcriptomic profiling and improve understanding of individual heterogeneity in response, supporting pregnancy-tailored precision medicine in sepsis.^78^

Maternal sepsis involves complex interactions across maternal, foetal, pathogen, environmental, and trimester-specific immune factors, complicating data interpretation. Machine learning and artificial intelligence (AI) enable integration of multi-omics, and clinical data to define reproducible immunophenotypes, earlier detection, predict disease trajectory and treatment responsiveness, and guide pregnancy-compatible immunomodulation.^74^ Time-series data further allows prediction of sepsis onset, clinical deterioration, and intervention risks. However, successful implementation requires biologically grounded endotypes, explainable models, and validation across diverse obstetric populations, accounting for different age, ethnic, geographical, BMI, and trimester subgroups,^79^ with prospective evaluation of maternal–foetal safety.

### Epigenetic reprogramming and long-term impacts

Sepsis-induced immune dysregulation reflects altered gene expression and chromatin states.^23^ Agents targeting histone deacetylases (HDAC), DNA methyltransferases (DNMT), and BET proteins offer potential therapeutic avenues to restore immune balance, but remain limited to preclinical or early-phase studies. Translation to pregnancy is constrained by safety concerns, as placental and foetal development depend on tightly regulated epigenetic programming,^61^ and evidence on pregnancy-specific pharmacokinetics (drug absorption, distribution, metabolism) and placental transfer is limited. Progress in this field requires pregnancy-specific models addressing foetal developmental timing, teratogenic risk, and offspring outcomes. Alternatives may include non-pharmacological epigenetic modulation (nutritional methyl donors),^75^ cell-type- or isoform-specific modulation rather than global DNMT or HDAC inhibition, and biomarker-guided strategies (e.g, DNA methylation) for patient stratification and real-time monitoring before systemic exposure. Long-term infant follow-up will be essential to assess transgenerational effects.

### Metabolic profiling and mitochondrial functions

Sepsis induces profound metabolic and bioenergetic dysregulation, including impaired glucose, lipid, and amino acid metabolism, and mitochondrial dysfunction.^63^ In maternal sepsis, interpretation of metabolic signatures is further complicated by trimester-specific adaptations that support maternal, foetal, and placental energy demands.^62^ Precision-medicine approaches can identify individual vulnerabilities through profiling of circulating metabolites such as acetate, succinate, and amino acids^67^ and mitochondrial respiration, revealing signatures of immune exhaustion or bioenergetic failure.^76^ Mitochondrial DNA variants may identify susceptibility to impaired oxidative phosphorylation. Targeted metabolic interventions, including supplementation with glutamine, arginine, or antioxidants,^75^ may enhance ATP production, limit oxidative stress, and restore mitochondrial function.

## Key lessons from pregnancy biology for future therapeutic directions of general sepsis

Pregnancy provides a natural model of immune recalibration, in which tolerance and defence are finely balanced to support foetal survival and pathogen protection. Balanced regulation of transcriptional programmes (NF-κB, JAK-STAT) and epigenetic markers; dynamic immune cell shifts (macrophages and T-cells); hormonal modulation (progesterone, oestrogen); vascular remodelling (VEGF–angiopoietin balance), and unique placental immune pathways together illustrate the capacity of pregnancy to maintain immune equilibrium.^36^^,^^38^^,^^59^ Translating these temporally staged mechanisms to non-pregnant sepsis may offer opportunities for phase-specific and precision therapies, in which timing and dosage will be critical to restoring immune homoeostasis without impairing host defence.

## Conclusion

This review outlines the intersections between sepsis and pregnancy across physiological, immunological, and regulatory domains, revealing opportunities to achieve equilibrium in both contexts. We emphasise the need for precision-based individualised approaches to fine-tune this balance to improve maternal outcomes. As sepsis in pregnancy presents a unique challenge, compounded by gestation-specific immune and physiological dynamics that complicate diagnosis, treatment, and long-term impacts, optimising outcomes requires a pregnancy phase-specific precision framework integrating dynamic immune monitoring, individualised risk assessment, and targeted recovery strategies. Preconception and antenatal, genomic and immune profiling can identify women at risk for tailored prevention; Labour and peripartum, rapid diagnostics support timely, personalised care; Postpartum, longitudinal immune tracking and targeted therapies may guide recovery and reduce long-term morbidity. Collectively, these strategies can support a proactive, data-driven model for managing maternal sepsis, aligned with the unique immunophysiology of pregnancy.

## Outstanding questions in maternal sepsis research

Key knowledge gaps remain in pregnancy-appropriate immunomodulation, and designing personalised interventions for maternal sepsis. Better understanding is needed of how peripheral and local immune responses shape outcomes across different infections in pregnancy. Pregnant women are often excluded from therapeutic trials, yet the RECOVERY trial for COVID-19 showed that carefully designed studies can safely inform pregnancy-specific guidance. Emerging phase-specific approaches including immunomodulatory, genomic, epigenetic, and microbiome-targeted interventions will require rigorous evaluation of effectiveness in reducing mortality and long-term morbidity. Integrating socio-demographic, biological and comorbidity data for sepsis-risk stratification is complex, requiring extensive validation. AI tools may help to distinguish sepsis from normal pregnancy physiology, but validated thresholds for clinical interventions are lacking. Global implementation of precision strategies must consider feasibility and cost, particularly in low-resource settings to improve maternal and perinatal outcomes equitably.

**Search strategy and selection criteria**

Data were identified through comprehensive searches of MEDLINE, PubMed, and Google Scholar, with additional articles from citation searching. Search terms included “sepsis”, “pregnancy”, “maternal sepsis”, “immunology”, “regulatory/signalling pathways”, “trimester”, and “precision medicine”, covering titles, abstracts, keywords, and subject headings. Of 2662 records retrieved, duplicates were removed and the remaining references were screened in Covidence. Sixty articles were included prioritising studies published in the past five years with an additional 19 from citation searching.

## Contributors

HEA: Conceptualisation, Literature search, Software, Data analysis, Generating figures and tables, Writing–Original Draft, Reviewing and Editing. MK: Conceptualisation, Funding acquisition, Supervision, Writing–Reviewing and Editing. JCK: Conceptualisation, Funding acquisition, Supervision, Writing–Reviewing and Editing. All authors read and approved the final version of the manuscript.

## Declaration of interests

JCK reports a grant to his institution from the Danaher Beacon Programme for work on RNA biomarker point-of-care test development in sepsis for endotype assignment. All remaining authors declare that they have no competing interests.
