# Supplementary material for: Immunology of pregnancy and sepsis: shared and specific pathways guiding future precision care
Source: eBioMedicine. 2026 Jul 11;130:106364. doi: 10.1016/j.ebiom.2026.106364 (PMC13382050; doi:10.1016/j.ebiom.2026.106364)
Supplement: Supplementary Table 1 [file mmc1.docx]

**Supplementary Data**

**Supplementary Table 1: Variation in cell counts across pregnancy trimesters and in sepsis, compared with non-pregnant reference levels.** Data are presented as mean cell counts (cells per microlitre of blood), with ranges shown in parentheses. Reference values were derived from published studies on normal pregnancy​^14​,​24–41​^ and sepsis in non-pregnant state.^9​,​21​,​23​,​24​,​28​,​30​,​33–35​,​38​,​42–51^​

| **Cell Type** | **Cellular concentrations (Cells/µL)** | | | | | |
| --- | --- | --- | --- | --- | --- | --- |
|  | **Normal**  **Non-pregnant** | **Pregnancy** | | | | **Sepsis**  **Non-pregnant** |
|  |  | **First**  **trimester** | **Second trimester** | **Third**  **trimester** | **Labour** |  |
| WBC (total) | 7,500  (4,000-11,000) | 8,500  (5,000-12,000) | 9,500  (6,000-13,000) | 10,750  (6,500-15,000) | 19,000  (9,000-29,000) | 17,000  (< 4,000  30,000) |
| Neutrophils | 4,750  (1,500–8,000) | 5,250  (2,500–8,000) | 6,000  (3,000–9,000) | 7,500  (4,000–11,000) | 8,500  (5,000–12,000) | 12,250  (1,500–23,000) |
| Basophils | 50  (0–100) | 50  (0–100) | 50  (0–100) | 50  (0–100) | 70  (40–100) | 10 |
| Eosinophils | 250  (0–500) | 200 | 200 | 200 | 115 | 50 |
| Monocytes  (total) | 500  (200–800) | 650  (200–1,100) | 650  (200–1,100) | 800  (200–1,400) | 850  (300–1,400) | 650  (100–1,200) |
| Classical monocytes (CD14++CD16–) | 239 | 286 | 280 | 338 | 340 | 500  (400–600) |
| Intermediate monocytes (CD14++CD16+) | 6·9 | 14·7 | 15·4 | 28 | 30 | 70  (40–100) |
| Non-classical monocytes (CD14+CD16++) | 13·5 | 15·2 | 14·2 | 13·1 | 13·1 | 10 |
| Lymphocytes  (total) | 2,800  (1,000–4,600) | 2,350  (1,100–3,600) | 2,400  (900–3,900) | 2,350  (1,100–3,600) | 2,000  (800–3,200) | 750  (500–1,000) |
| T regulatory cells | 66.9 | 133 | 120 | 100 | 60 | 35  (27–43) |
| CD4 T cells | 850  (500–1,200) | 600  (545–655) | 596·5  (540–653) | 591  (536–646) | 646 | 255  (90–420) |
| CD8 T cells | 525  (150–900) | 455 | 455 | 447 | 450 | 180  (60–300) |
| B cells | 296 | 178 | 174 | 160 | 110 | 88 |
| Peripheral  Natural Killer cells | 275  (150–400) | 232·6 | 183·4 | 166·9 | 166·9 | 75  (50–100) |
| Myeloid  Dendritic cells | 18·6 | 23·9 | 21·9 | 16·2 | 10 | 4·2 |
| Plasmacytoid Dendritic cells | 8·3 | 7·9 | 5·9 | 5·8 | 5 | 1·9 |
| Platelets | 300,000 (150,000–450,000) | 285,000 (150,000–420,000) | 275,000 (140,000–410,000) | 265,000 (130,000–400,000) | 280,000  (140,000–420,000) | 85,000  (20,000–150,000) |

Heterogeneity in cell count data can arise due to factors such as cohort characteristics (e.g., geography, body mass index, and parity), timing of sampling (e.g., labour stage), measurement methods (e.g., bead-based absolute counts, complete blood count-derived estimates, flow cytometry gating strategies, and relative expansions of specific cell subsets), and disease context (e.g., type of sepsis pathogen and disease progression). Despite this variability, the cell count ranges presented here were derived from a comprehensive literature review of representative, peer-reviewed sources. As such, these findings provide a broad reflection of cell composition and counts during advancing pregnancy and in sepsis.
